# Constitutive and inducible fibrosis explain immune variation among threespine stickleback populations

**DOI:** 10.64898/2026.04.27.721125

**Published:** 2026-05-04

**Authors:** Emma S. Choi, Ben A. Flanagan, Heather Alexander, John Berini, Alex Yeung, Cole J. Wolf, Viola Watts, Grace Vaziri, Nataly Vargas, Caroline Szajada, Penelope Steffen, Ipsita Srinivas, Mehreen Shahid, Ana Santacruz, Kaligua Rochon, Luke Rippin, Edith Reyes Contreras, Emma Redfield, Emma Polard, Cate Patterson, Fahad Gilani, Julian Flanagan, Shira Dubin, Peaches Cooper, Panna Codner, Amy Chen, Gwen Casey, Abigail G. Albright, Jessica Hite, Jesse N. Weber, Daniel I. Bolnick, Amanda K. Hund

**Affiliations:** 1 Department of Ecology and Evolutionary Biology, University of Connecticut, 75 North Eagleville Rd, Storrs CT 06269 USA; 2 Department of Biology, Carleton College, Northfield, Minnesota, USA; 3 Bamfield Marine Sciences Centre, Bamfield, British Columbia, Canada, V0R 1B0; 4 Stowers Institute for Medical Research, Kansas City, MO, USA; 5 Department of Pathobiological Science, School of Veterinary Medicine, University of Wisconsin-Madison, Madison, Wisconsin, USA; 6 Department of Integrative Biology, University of Wisconsin-Madison, Madison, Wisconsin, USA; 7 University of Victoria, Victoria, British Columbia, Canada; 8 University of British Columbia, Vancouver, British Columbia, Canada; 9 Current address: Institute for Integrative Cell Biology and Physiology, University of Münster, Münster, Germany.; 10 Current address: University of Minnesota; 11 These authors contributed equally to this work; 12 Lead contact

**Keywords:** host-parasite interactions, adaptation, ecoimmunology, evolution, genetic variation, *Schistocephalus solidus*

## Abstract

Population-specific immunity can drive variation in infection outcomes, but studying immune variation in the wild is challenging because exposure histories are unknown. Comparing wild populations with those reared in a common environment can disentangle genetic and environmental drivers of immunity. We applied this approach in freshwater threespine stickleback, where populations vary in their use of intraperitoneal fibrosis to defend against the helminth parasite *Schistocephalus solidus*. We combined a 46-lake immune survey with a common garden experiment using 20 representative populations to examine variation in fibrosis and infection. Laboratory assays included exposures to live tapeworms and immune challenges with tapeworm proteins and aluminum phosphate (Alum). We found heritable variation in both constitutive fibrosis and inducible fibrosis. Inducible responses to tapeworms were associated with lake environmental conditions, with fish from more eutrophic-like lakes showing stronger fibrosis induction than those from more oligotrophic-like lakes. Together, these results show how integrating wild immune variation with common garden experiments can reveal novel heritable defenses and link their evolution to ecological variation.

## Introduction:

Genetic variation in immunity provides the raw material for host defense evolution and contributes to variation in parasite susceptibility among individuals and populations (Brodin & Davis, 2016; Fumagalli et al., 2011; Sanz et al., 2018; Stevens et al., 2023). Because parasite-driven selection can be exceptionally strong, immune genes are often highly polymorphic and rapidly evolving (Fortier & Pritchard, 2025; Haldane, 2006). However, immune variation observed in the wild does not necessarily reflect host adaptive evolution, and may instead arise from phenotypic plasticity, parasite genetic variation, synergistic effects of host and parasite genotypes, or interactions between genotype and the environment. These factors complicate interpretation of immune trait variation in natural populations.

Common-garden experiments combined with controlled immune challenges can partition genetic and environmental sources of immune variation by minimizing environmental effects and revealing heritable differences among hosts. When paired with data from wild populations, this approach can link immune traits to ecological conditions and potential selective pressures. However, relatively few studies integrate common-garden experiments with broad sampling of wild populations (Bolnick et al., 2025; Sasser & Weber, 2023). Here, we apply this framework to threespine stickleback, focusing on fibrosis-based resistance to a common tapeworm.

Threespine stickleback (*Gasterosteus aculeatus*) and the tapeworm *Schistocephalus solidus* form a well-studied host–parasite system in which freshwater populations vary in their resistance to infection. Following colonization of freshwater environments by marine ancestors, stickleback populations evolved increased resistance to this parasite (Sriramulu et al., 2025; Weber et al., 2017), but now differ markedly in immune responses across lakes (Bolnick et al., 2026; Fuess et al., 2026; Hund et al., 2022; Weber et al., 2022). One key defense is peritoneal fibrosis, a conserved immune response that can encapsulate and restrict tapeworm growth (Vrtílek & Bolnick, 2021; Weber et al., 2022). Fibrosis reduces parasite success but also imposes fitness costs (De Lisle & Bolnick, 2021; Matthews et al., 2023), leading to variation among populations in both the presence and magnitude of this response. As a result, some populations exhibit strong fibrosis while others show reduced or absent responses, differences that are observed in both wild and laboratory settings (Bolnick et al., 2026; Hund et al., 2022; Weber et al., 2022). However, it remains unclear to what extent this natural variation in fibrosis is evolved versus plastic, and whether it is an adaptive response to any environmental variables.

Population-level variation in fibrosis could arise from multiple sources. First, differences may reflect environmental plasticity if populations experience different exposure rates to *S. solidus*, such that fibrosis is induced more frequently or strongly in high-exposure environments; in this case, population differences should disappear under common-garden conditions. Second, variation could be driven by parasite genetic differences, as distinct tapeworm genotypes present different antigens (Wang & Bolnick, 2025) and can elicit variable immune responses in stickleback (Bolnick et al., 2024; Collias et al., 2025; Scharsack et al., 2013), potentially leading to differences in downstream fibrosis induction depending on the tapeworm genotype encountered. Finally, differences may reflect host genetic divergence, with populations evolving differences in baseline or inducible fibrosis. Constitutive differences should persist in laboratory-reared fish regardless of immune challenge, whereas inducible variation should appear as differences in response to parasite exposure (G×E), potentially modified by host–parasite genotype combinations (G×G).

If fibrosis is heritable, an important question is whether variation is adaptive. Because fibrosis imposes both costs and benefits, its optimal level may differ among populations depending on local conditions (Graham et al., 2005; Lazzaro & Little, 2009). Stickleback diets vary across lakes: some consume primarily mid-water, planktonic prey (“limnetic”), while others feed mainly on bottom-associated prey (“benthic”) (Bolnick et al., 2003; Bolnick & Ballare, 2020; Snowberg et al., 2015). Because *S. solidus* is transmitted via planktonic copepods, limnetic-feeding populations are likely exposed to higher parasite loads, potentially selecting for stronger fibrosis, whereas benthic-feeding populations may experience weaker selection (Stutz et al., 2014). Alternatively, population differences could reflect non-adaptive processes, such as historical contingency, founder effects, bottlenecks, or mutation accumulation. By linking population-level fibrosis to ecological factors, we can test whether heritable variation corresponds with selective pressures arising from lake-specific environments.

To determine the genetic and ecological drivers of population-level immune variation, we conducted a 46-lake survey of fibrosis in wild stickleback populations. From this, we selected 20 populations spanning the observed range of fibrosis and reared them in a common garden, exposing fish to multiple *S. solidus* genotypes, including local and non-local parasites. This design allowed us to test whether population differences persist under uniform conditions, whether they depend on tapeworm genotype, and whether laboratory responses covary with lake ecology. We found that fibrosis variation is heritable, with both constitutive and inducible components, whereas tapeworm genotype had only a weak effect. Inducible responses in the laboratory were correlated with lake ecology, though in an unexpected direction. Overall, this study combines broad population sampling with controlled experimental exposures to disentangle the roles of host evolution, parasite variation, and ecological context in shaping immune variation.

## Results:

### Population differences in infection prevalence and fibrosis in nature

**Figure 1:**
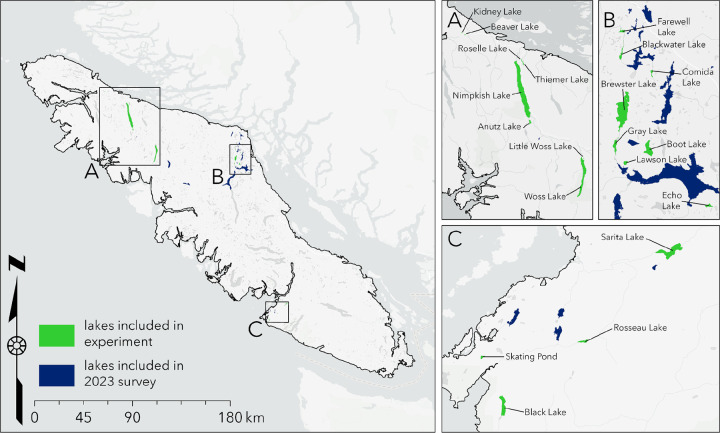
Map of lakes included in the 2023 survey and in the experiment. Lakes included in the experiment were also included in the 2023 survey.

**Figure 2:**
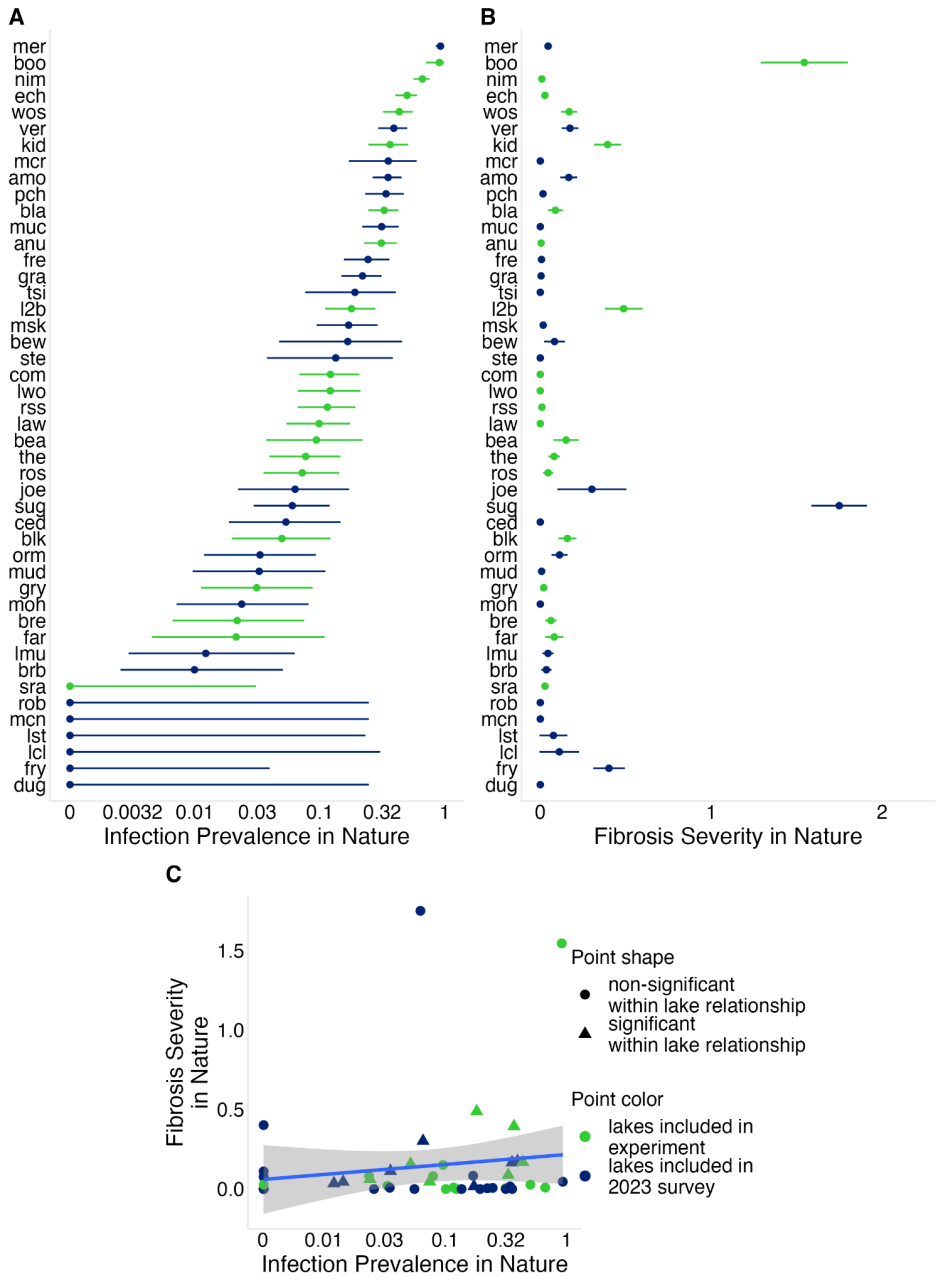
Population-level variation in infection prevalence and fibrosis severity in nature. (A) Infection prevalence across populations;points represent the proportion of infected fish in each population, with 95% binomial confidence intervals. (B) Mean fibrosis severity across populations; points represent population means and error bars show standard errors. (C) Relationship between infection prevalence and fibrosis severity across populations; points represent population means. Triangles indicate populations in which infection and fibrosis are significantly correlated among individual fish (P < 0.05). Infection prevalence in (A) and (C) is plotted on a log scale, with axis labels shown on the original (unlogged) scale. Lake abbreviations correspond to names and sample sizes in Supplementary Table 1.

To determine population-level variation in fibrosis severity and *S. solidus* infection prevalence, we conducted an ecological and immunological survey of 46 Vancouver Island lake stickleback populations in 2023 (Figure 1; Table S1). Infection prevalence varied widely among lakes, ranging from 0% to 93% across populations (Figure 2A). A binomial GLMM estimated mean prevalence at 10.5%, with strong among-population variation (ICC = 0.49), indicating nearly half the variation in infection was attributable to lake identity.

Fibrosis severity also differed among populations (Figure 2B). Because fibrosis was scored on an ordinal 0–4 scale, we analyzed variation using Bayesian ordered logistic regression, treating population as a random effect. In this model, the among-population standard deviation $\left(\sigma_{\text{pop}}\right)$ measures variation in lake means, and values substantially greater than zero indicate population divergence in fibrosis. This revealed strong among-population variation $\left(\sigma_{\text{pop}}=1.93[1.41,2.65]\right)$, with 22% of variance attributable to lake differences. A linear model gave similar support (F_45_,_1532_ = 19.12, P < 0.0001). Sugsaw (sug) and Boot (boo) lakes showed the highest mean fibrosis (1.75, se = 0.16; 1.55, se = 0.25, respectively), whereas 12 lakes showed no visible fibrosis.

Lakes with higher log *S. solidus* prevalence tended to have higher fibrosis, but the relationship was weak and non-significant across lakes (r = 0.14, P = 0.37; Figure 2C), consistent with previous results from Alaska (Bolnick et al., 2026). By contrast, within lakes, infected individuals had higher fibrosis than uninfected fish (lco = 1.82 [1.19, 2.35]), consistent with previous laboratory and field studies showing infection-associated induction of fibrosis (Weber et al., 2022). The magnitude of this effect varied among populations $\left(\sigma_{\text{pop}}=0.71[0.18,1.39]\right)$, indicating that lake identity modulates the strength of the infection–fibrosis relationship. However, this variation in infection-associated fibrosis explains only a fraction of the among-lake variation in fibrosis severity.

### Common garden experiment: heritable variation in fibrosis

To test whether fibrosis responses differ among populations, we conducted a common-garden experiment using stickleback from 20 Vancouver Island populations spanning the range of infection prevalence and fibrosis severity observed in the wild (Figure 2). We raised embryos under controlled laboratory conditions and later exposed adult fish to immune challenges (alum injection, tapeworm protein injection, and live tapeworm ingestion) to quantify population differences in fibrosis response.

#### Populations differ in constitutive fibrosis

**Figure 3:**
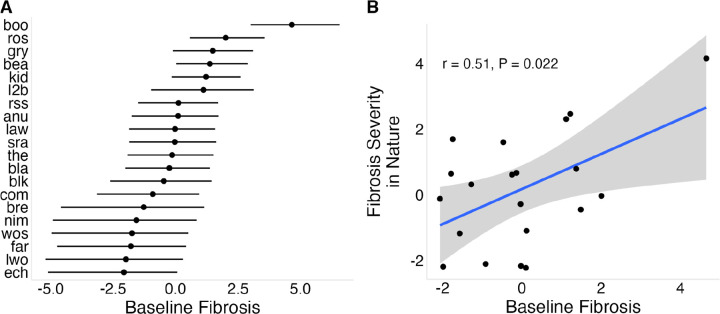
Population-level variation in baseline fibrosis and its relationship to wild fibrosis. (A) Baseline fibrosis across populations; points represent Bayesian estimates for uninjected control fish, standardized such that 0 represents the mean across populations. (B) Relationship between Bayesian estimates of baseline fibrosis measured in the laboratory and fibrosis severity observed in wild populations.

We observed population differences in baseline (constitutive) fibrosis in lab-reared stickleback that did not receive immune stimulation (Figure 3A). In the uninjected controls, fibrosis was generally low (mean of 0.44), but showed substantial among-population variation ($\sigma_{\text{pop}}=1.89[1.15,2.91]$; Supplementary Data 2, Panel D), indicating heritable differences in constitutive fibrosis. Five populations showed no fibrosis, whereas Boot Lake (boo) showed the highest mean fibrosis (2.30, se = 0.169). Fibrosis in PBS-injected controls was highly correlated with uninjected fish across populations (r = 0.76, P = 0.0001), indicating minimal effect of injection itself. Mean fibrosis in the wild was positively associated with baseline fibrosis in the lab from the same populations (Figure 3B; r = 0.51, P = 0.022), although this relationship weakened after excluding Boot Lake (Figure S2; r = 0.35, P = 0.12).

#### Populations differ in their fibrosis response to an adjuvant (alum)

**Figure 4:**
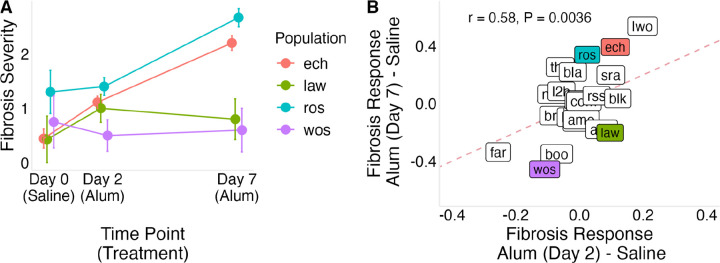
Temporal dynamics of fibrosis responses to alum injection across populations. (A) Fibrosis severity in saline-injected controls (day 0), and alum-injected fish at two and seven days post-injection, shown for four representative populations. (B) Fibrosis response Bayesian effect sizes (alum-injected compared to saline control) at two and seven days post-injection; points represent population-level responses. Colored points correspond to the populations highlighted in (A): Roselle Lake (ros) shows a stronger increase from day 2 to day 7, Echo Lake (ech) shows similar responses at both time points, Lawson Lake (law) shows a rapid early response with little subsequent increase, and Woss Lake (wos) shows minimal change. The dashed line indicates equal responses at day 2 and day 7.

To determine if populations vary in the rate of fibrosis induction following immune stimulation, we injected lab-reared fish with alum, a known fibrosis-inducing stimulus (Fuess et al., 2026; Hund et al., 2022; Vrtílek & Bolnick, 2021), and measured responses at 2 and 7 days post injection (dpi). At 2 dpi, alum injection increased fibrosis relative to saline controls (lco = 1.07 [0.60, 1.53], Supplementary Data 2, Panel E), but there was no detectable variation among populations in this response ($\sigma_{\text{pop}}=0.3[0.02,0.84]$, Supplementary Data 2, Panel F). By 7 dpi, the effect of alum injection was stronger (lco = 2.46 [1.88, 3.02], Supplementary Data 2, Panel G), and among-population variation in response increased as well ($\sigma_{\text{pop}}=0.45[0.02,1.18]$, Supplementary Data 2, Panel H). Using both timepoints in a combined model, we estimated the per-day increase in the odds of being in a higher fibrosis category following alum injection, which revealed both a strong overall effect of alum injection (lco = 0.42 [0.33, 0.50], Supplementary Data 2, Panel I) and significant among-population variation in the rate of response ($\sigma_{\text{pop}}=0.11[0.03,0.22]$, Supplementary Data 2, Panel J). This indicates that populations differ in the speed of fibrosis induction following immune stimulation. Representative populations showed distinct temporal trajectories of fibrosis induction (Figure 4A).

#### Tapeworm genotype affects fibrosis during live infection but not protein injection

To test whether tapeworm genotype influences host fibrosis responses, we injected lab-reared fish with proteins derived from geographically local and distant S. solidus populations. Injection with tapeworm protein produced a weak overall effect on fibrosis (lco = 0.33 [-0.11, 0.75], Supplementary Data 2, Panel K), but there was moderate evidence for among-population variation in response ($\sigma_{\text{pop}}=0.47[0.06,1.05]$, Supplementary Data 2, Panel L), indicating population differences in sensitivity to tapeworm antigens. There was no overall effect of tapeworm genotype on fibrosis (lco = 0.07 [-0.37, 0.48], Supplementary Data 2, Panel M), and only moderate support for among-population variation in differential responses to local and foreign tapeworm proteins (i.e. a G×G effect; $\sigma_{\text{pop}}=0.33[0.03,0.89]$, Supplementary Data 2, Panel N). Nevertheless, responses to locally and geographically distant tapeworm proteins were strongly correlated across populations (r = 0.58, P = 0.007), suggesting limited divergence in antigen-specific induction.

**Figure 5:**
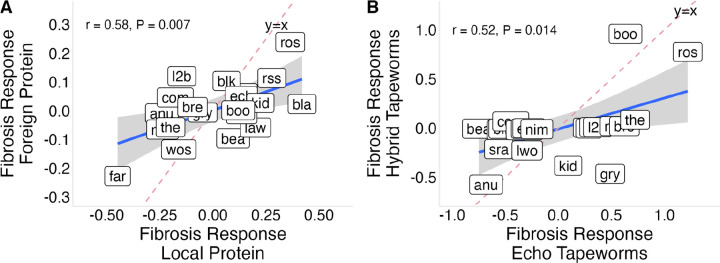
Population-level fibrosis responses to local versus foreign tapeworm exposures. (A) Fibrosis responses to local versus foreign tapeworm protein injection across populations; points represent population-level Bayesian posterior estimates. Responses are positively correlated, indicating no consistent effect of tapeworm genotype in protein exposure. (B) Fibrosis responses to live Echo versus hybrid tapeworm exposure across populations; points represent population-level Bayesian posterior estimates. Responses are not correlated across populations, and Bayesian ordinal logistic regression supports an effect of tapeworm genotype during live infection.

To test whether tapeworm genotype effects extend across broader geographic scales, we experimentally exposed lab-reared stickleback to live *S. solidus* tapeworms derived from either a local Vancouver Island population (Echo Lake) or hybrid tapeworms combining local and geographically distant lineages (Echo Lake and Skogseidvatnet Lake, Norway), providing a genetically divergent “foreign-like” comparison. Lab-raised fish exposed to native Echo Lake tapeworms showed lower fibrosis than fish exposed to hybrid tapeworms (Echo - hybrid lco = −0.85 [−1.89, −0.07], Supplementary Data 2, Panel O). Although the magnitude of this genotype effect varied among stickleback populations ($\sigma_{\text{pop}}=1.09[0.25,2.22]$, Supplementary Data 2, Panel P), responses to Echo and hybrid tapeworm exposure were positively correlated across populations (r = 0.52, P = 0.014; Figure 5B) indicating that populations differ primarily in the strength, rather than the direction, of their responses to tapeworm genotype.

Interpretation of genotype effects in live infections is limited by differences in exposure intensity between treatments: Fish were exposed to live tapeworms via infected copepods, but the infection rate in copepods differed between treatments, leading to higher effective exposure to Echo Lake tapeworms. This difference would be expected to produce stronger fibrosis in response to Echo tapeworms. However, we instead observed higher fibrosis following exposure to hybrid tapeworms that include foreign genotypes from Europe. This pattern provides further support for an effect of tapeworm genotype on host immune activation, although interpretation remains limited by unequal exposure intensity between treatments. Given these differences, subsequent analyses focus on fish exposed to Echo Lake tapeworms.

#### Heritable variation in inducible fibrosis responses among stickleback populations

**Figure 6:**
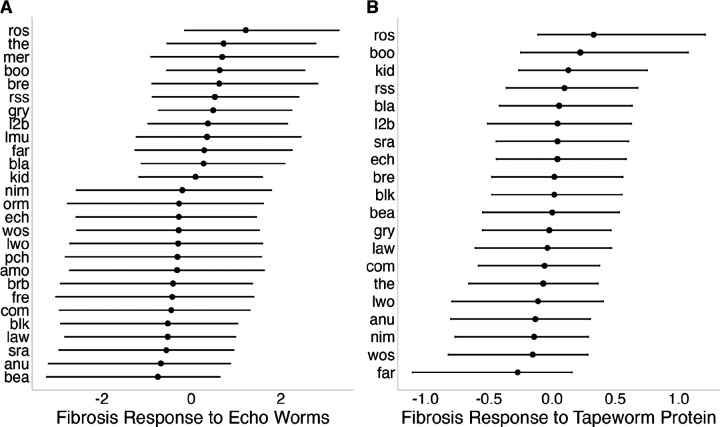
Population-level variation in fibrosis responses to tapeworm exposure. (A) Fibrosis response to live Echo Lake tapeworm exposure; points represent population-level Bayesian posterior means and error bars show 95% credible intervals. (B) Fibrosis response to tapeworm protein injection; points represent population-level Bayesian posterior means and error bars show 95% credible intervals.

To characterize heritable variation in fibrosis response among stickleback populations, independent of tapeworm genotype effects, we used results from the live tapeworm exposures and tapeworm protein injections described above. For live Echo tapeworm exposures, infection success was low across populations (5.6% [3.17%, 9.06%]), consistent with these hosts having low susceptibility to exposure. Stickleback exposed to Echo Lake tapeworms did not show elevated fibrosis relative to unfed controls, with a weak overall trend in the opposite direction (lco = −0.77 [−1.85, 0.2], Supplementary Data 2, Panel Q). Despite this, there was support for a population by exposure interaction effect ($\sigma_{\text{pop}}=0.92[0.06,2.18]$, Supplementary Data 2, Panel R), indicating that populations differ in their response to tapeworm exposure, reflected in the distribution of Bayesian estimates of population-specific responses to tapeworm exposure (Figure 6A). A similar pattern was observed for hybrid tapeworm exposure, which showed no overall effect on fibrosis (lco = 0.18 [-0.61, 0.92], Supplementary Data 2, Panel S), but comparable among-population variation in response ($\sigma_{\text{pop}}=0.91[0.25,1.58]$, Supplementary Data 2, Panel T). Although exposure effects for individual populations were highly uncertain, both assays consistently indicated non-zero among-population variation in responsiveness to infection.

Because live tapeworm exposures integrate multiple processes (e.g. infection success, parasite growth, immune activation by the host, and immune suppression by live tapeworms), we used tapeworm protein injections to isolate antigen-driven fibrosis responses. Tapeworm protein produced a modest overall increase in fibrosis (lco = 0.38 [-0.04, 0.78], Supplementary Data 2, Panel K), with evidence for among-population variation in response ($\sigma_{\text{pop}}=0.25[0.02,0.66]$, Supplementary Data 2, Panel L), reflected in a broad distribution of Bayesian estimates of population-specific responses to tapeworm protein injection (Figure 6B). Raw mean differences alongside population-level Bayesian estimates (with credible intervals) are provided in Table S2 to aid interpretation of population-specific responses, which ranged from negligible to strong induction. Protein- and live-exposure responses were positively correlated (r = 0.48, P = 0.03; Figure S3C), suggesting partially shared underlying sensitivity to *S. solidus* antigens.

To test whether laboratory immune phenotypes predict fibrosis in wild populations, we related Bayesian estimates of population effects for wild fibrosis to corresponding estimates of constitutive and inducible (population × treatment) fibrosis responses measured in the laboratory. Constitutive fibrosis explained 26% of variation in wild fibrosis estimates, compared to 15% for tapeworm protein responses and 11% for alum responses. However, these relationships were not robust to exclusion of Boot Lake (boo); without Boot, explained variance dropped to ~4–6% across predictors, indicating that cross-population associations were strongly influenced by a single high-leverage population.

Population constitutive fibrosis was positively correlated with inducible responses to tapeworm protein (r = 0.70, t = 4.20, P = 0.0006), but not with alum responses at day 2 (r = −0.05, t = −0.21, P = 0.84) or day 7 (r = −0.23, t = −0.99, P = 0.34). This suggests that constitutive fibrosis is associated with responsiveness to tapeworm antigens, but not to a general inflammatory stimulus, consistent with previous results from Alaska (Bolnick et al., 2026). By contrast, inducible responses were only weakly and non-significantly correlated across challenges (tapeworm protein vs alum day 2: r = 0.25, t = 1.11, P = 0.28; tapeworm protein vs alum day 7: r = 0.26, t = 1.14, P = 0.27), indicating that constitutive and inducible fibrosis are partially coupled in response to tapeworm-derived stimuli, but not consistently coordinated across immune challenges.

We also tested whether laboratory assays can explain among-lake variation in fibrosis-infection associations observed in the wild - that is, the extent to which infected fish show elevated fibrosis relative to uninfected fish within a lake. Although this association was consistently positive, its strength varied among populations. We asked whether this variation could be explained by Bayesian population-specific estimates of fibrosis responsiveness measured in the laboratory, including responses to tapeworm protein, live tapeworm exposure, and alum injection. Variation in wild fibrosis–infection associations showed a weak positive, though non-significant, relationship with laboratory responses to tapeworm protein (r = 0.290, t = 1.28, P = 0.218), and was uncorrelated with responses to alum or Echo Lake tapeworm exposure (both P > 0.5). Responses to Canada–Norway hybrid exposure showed a marginal positive correlation (r = 0.42, t = 2.09, P = 0.05), but this pattern was driven by a small number of influential populations and was not considered robust evidence that laboratory assays explain among-lake variation in fibrosis–infection associations.

### Inducible fibrosis responses vary with lake ecology

To determine whether lake ecology influences heritable fibrosis, we related ecological covariates (lake depth, lake surface area, surface temperature, dissolved oxygen, chlorophyll *a*, and pH) to constitutive fibrosis and inducible fibrosis responses to tapeworm protein. Environmental variation was summarized using principal component analysis; PC1 (58.6% of variance) reflected a trophic gradient, with lower values corresponding to deeper, larger lakes with higher dissolved oxygen and pH, and lower chlorophyll *a* concentrations, while surface temperature contributed relatively little to this axis (Table S3).

**Figure 7:**
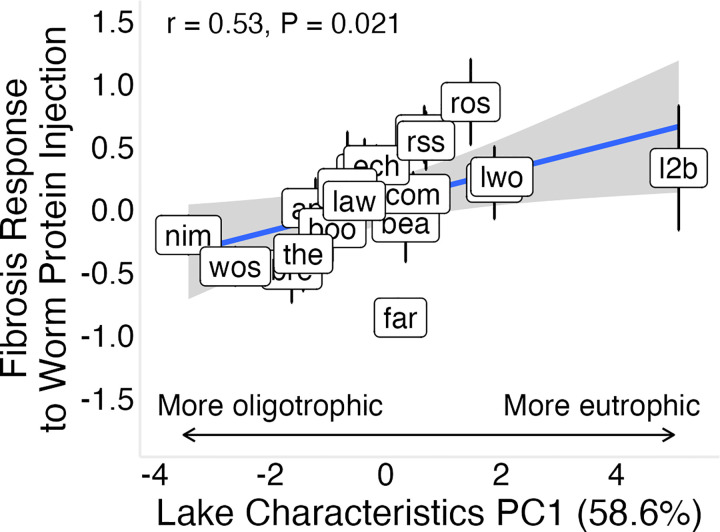
Association between environmental variation and inducible response to tapeworm protein. Fibrosis response to tapeworm protein injection is positively associated with lake environmental PC1 across populations. Response is calculated as the raw difference in fibrosis score between tapeworm protein–injected and control fish. PC1 represents a trophic gradient, separating more oligotrophic lakes (deeper, higher oxygen, lower chlorophyll *a*) from more eutrophic lakes (shallower, lower oxygen, higher chlorophyll *a*). A similar but weaker positive trend is observed using Bayesian population-level estimates (Figure S3).

Baseline fibrosis showed no significant association with environmental PC1 (58.6% of variance explained; Figure S5A; r = 0.16, P = 0.51), but this relationship was sensitive to population composition: excluding Boot Lake yielded a positive trend (Figure S5; r = 0.45, P = 0.06), indicating dependence on a high-leverage population. In contrast, inducible responses showed a clearer association with environmental variation (Figure 7; r = 0.53, P = 0.02), which was qualitatively consistent but attenuated in Bayesian estimates due to shrinkage (Figure S4; r = 0.35, P = 0.14). Individual covariates showed similar patterns, with lake depth, area, and pH negatively associated with inducible fibrosis (P < 0.05, Figure S6). Together, these results suggest that lake ecology is more consistently associated with inducible than constitutive fibrosis, with stronger inducible responses in more eutrophic systems.

## Discussion:

To determine the sources of immune variation among wild stickleback populations, we conducted a common garden experiment designed to distinguish among three competing hypotheses: 1. populations differ in their exposure to tapeworms, leading to plastic differences in fibrosis; 2. populations encounter genetically distinct tapeworm variants that induce different degrees of fibrosis; or 3. populations exhibit heritable differences in their fibrosis expressed either constitutively (all the time), or inducibly (in response to tapeworm infection). We found strongest support for heritable immune differences among populations, with limited support for plastic or parasite genotype–dependent explanations. Previous work identified genetic differences in fibrosis among two or three populations (Hund et al., 2022; Weber et al., 2022); our results generalize those findings and additionally suggest constitutive variation contributes to population differences, although this signal was partly driven by a high-influence population (boo). Notably, constitutive fibrosis was not consistently associated with ecological variation, whereas inducible responses showed clearer alignment with lake-specific ecological conditions.

### Little support for non-genetic explanations for fibrosis variation

If population-level variation in fibrosis were driven solely by differences in tapeworm exposure, we would expect minimal among-population differences under controlled laboratory conditions. Instead, substantial variation in fibrosis persisted among populations reared without immune stimulation as well as those exposed to alum, tapeworm proteins, and live tapeworms. This suggests that exposure-driven plasticity alone does not fully account for observed population differences in the wild.

A second non-genetic explanation is that variation reflects differences in tapeworm genotype across populations. Under this scenario, we would expect consistent population-specific responses to local versus foreign tapeworm antigens, and for fibrosis patterns in the field to correspond to responses induced by sympatric parasites. However, this expectation is not supported within Vancouver Island: populations from the same geographic region, likely exposed to similar tapeworm genotypes due to bird-mediated dispersal (Shim et al., 2022), exhibited marked differences in fibrosis, and tapeworm genotype had no detectable effect on among-population variation. Together, these results suggest that neither exposure history nor parasite genetic variation can fully explain the observed population-level differences at this spatial scale.

At broader geographic scales (North America vs Europe), however, parasite genotype did influence responses: fibrosis was lower following exposure to local Echo parasites than to hybrid worms. This pattern is consistent with local parasites evading or suppressing host immunity, whereas foreign parasites are more readily detected, echoing a “resist globally, infect locally” dynamic. (Weber et al., 2017).

### Constitutive genetic differences in fibrosis among lake populations

Fibrosis differed among stickleback populations raised in a common lab setting, confirming fibrosis variation is highly heritable. We find evidence that this variation occurs both in constitutive fibrosis and in the magnitude of inducible responses to immune challenge. Fibrosis varied extensively among populations in control fish (i.e., unexposed or saline-injected), suggesting heritable differences in constitutive fibrosis among populations. These differences were broadly consistent with variation observed in wild-caught fish, although the strength of this correspondence was influenced by a single high-leverage population. These results are notable because stickleback fibrosis has generally been considered primarily inducible, activated in response to immune stimulation (Bolnick et al., 2024; Hund et al., 2022; Vrtílek & Bolnick, 2021; Weber et al., 2022).

This raises the question of why some populations exhibit constitutive fibrosis despite its known fitness costs. Elevated constitutive fibrosis may be favored under high *S. solidus* pressure if it reduces the costs of repeated inflammatory responses (Paludan et al., 2021), if populations evolve to overshoot optimal defenses (Urban et al., 2013), or if it provides protection against a broader suite of parasites (Dezfuli et al., 2015; Guagliardo et al., 2019). However, constitutive fibrosis observed in the laboratory does not always match patterns in wild populations; some populations exhibited elevated constitutive fibrosis under laboratory conditions despite showing little to no fibrosis in wild-caught individuals. One explanation is that laboratory environments, with reduced pathogen exposure and altered microbial communities, may induce hyper-reactive immune states and elevated baseline fibrosis (Donald & Finlay, 2023; McDade et al., 2016). Together, these results suggest that constitutive fibrosis reflects both historical selection and environmental context rather than a single adaptive strategy.

### Genetic variation in inducible fibrosis responses

Fibrosis also differed between control fish and those challenged with immune stimuli (alum, live tapeworms, or tapeworm protein). The response to alum was especially strong, consistent with the activation of an evolutionarily ancient pathway predating jawed vertebrates (Vrtílek & Bolnick, 2021). Responses to live tapeworms and tapeworm protein were generally weaker, although we still observed among-population variation in laboratory settings, consistent with heritable differences in inducible fibrosis (GxE interactions).

Tapeworm-induced fibrosis explains only part of the variation in wild populations but is associated with ecological conditions. We expected inducible fibrosis to reflect alternative defense strategies, including unresponsiveness in naïve marine populations (Ayres & Schneider, 2012; Hund et al., 2022; Vrtílek & Bolnick, 2021; Weber et al., 2022), inducible resistance (Ayres & Schneider, 2012; Hund et al., 2022; Weber et al., 2022), or tolerance via suppression of fibrosis (Weber et al., 2022). The predominance of weak responses could therefore reflect tolerance strategies, limited evolution of inducible resistance after colonization, or both.

In the Nimpkish and Anutz Lake populations, stickleback morphologically resemble marine fish, retaining extensive armor plating (Yeung and Bolnick, unpublished). Because armor plate reduction is a rapid and well-characterized marker of freshwater adaptation (Colosimo et al., 2004; Roberts Kingman et al., 2021), the persistence of marine-like morphology in these populations suggests limited freshwater adaptation likely due to ongoing gene flow with marine populations. These marine-like populations fail to mount fibrotic responses following tapeworm exposure and show limited constitutive fibrosis. This result confirms previous inferences that marine stickleback lack fibrosis (Bolnick et al., 2026; Weber et al., 2022).

At the other extreme, low inducible fibrosis in some populations is consistent with tolerance. In Gosling Lake, tolerance is associated with a deletion in the immune regulatory gene *spi1b,* reducing fibrosis potential and pro-fibrotic activity (Fuess et al., 2021; Weber et al., 2022). However, tolerance may not always be favored: immune-capable immigrant genotypes nearly replaced Gosling Lake ancestry within ~10 generations, suggesting costs of tolerance even in locally adapted populations (Flanagan et al., 2025). These contrasting outcomes indicate that alternative defense strategies are dependent on ecological context which is substantiated by our observation that reaction norms are related to ecological covariates.

### Environmental and ecological drivers of the fibrosis reaction norms

The strong relationship between the inducible fibrosis response and environmental conditions indicate this type of response plays an outsized role in the eco-evolutionary dynamics of tapeworm defenses (Figure S10). Fibrosis responses were strongest in smaller, shallower lakes with higher chlorophyll *a* and lower dissolved oxygen (environmental PC1; Figure 7), providing the first direct evidence linking immune variation to lake ecology.

Stickleback diet varies seasonally and is associated with ecomorph, with limnetic fish generally consuming more copepods and often carrying higher parasite burdens within lakes (Reimchen, 1982, 1997; Schluter & McPhail, 1992; Stutz et al., 2014). However, across lakes, benthic populations exhibit higher average infection loads than limnetic populations, a countergradient pattern attributed to stronger immunity in limnetic fish (Stutz et al., 2014). Consistent with this, stickleback from benthic zones tend to have higher tapeworm abundance than those from limnetic zones (Arostegui et al., 2018).

One explanation for our results is that benthic populations may consume fewer copepods overall but encounter more heavily infected copepods, leading to higher effective exposure in smaller, oligotrophic lakes. Alternatively, limnetic populations may experience higher encounter rates but evolve tolerance, although this would predict higher infection prevalence than we observe. More plausibly, inducible fibrosis is favored in oligotrophic lakes where copepods, though a smaller dietary component, are more likely to carry infection.

Supporting this, tapeworm eggs deposited by aquatic birds hatch preferentially in shallow, warm, sunlit waters (Dubinina, 1966), increasing effective transmission in shallow lakes by expanding the spatial extent of suitable habitat and concentrating hosts, parasites, and intermediate hosts. These processes likely increase encounter rates between stickleback and infected copepods in shallow systems, elevating selection for inducible fibrosis. Although lake ecology could directly influence immunity, our results more strongly support an indirect mechanism mediated by parasite exposure through the copepod community.

## Conclusion

To capture the full spectrum of immunological diversity in nature, it is important to study many populations. Laboratory studies often focus on limited genetic variation, obscuring natural heterogeneity in immune traits and their ecological and evolutionary drivers (Graham, 2021).Similarly, studies restricted to a small number of populations may fail to capture the extent of immune trait variation (e.g. Weber et al 2022). By integrating field surveys with controlled laboratory challenges across many populations, we linked genetic and environmental variation to immune phenotypes in wild stickleback.

Wild populations exhibited substantial variation in fibrosis, but without controlled exposure it is difficult to distinguish whether populations are naive, tolerant, inducibly-resistant, or constitutively-resistant. Our results reveal three main patterns. First, baseline fibrosis, reflecting constitutive resistance, explains substantial among-population variation in common garden conditions, highlighting a previously underappreciated axis of immune differentiation. Second, some populations consistently lacked fibrosis, consistent with tolerance and with limited resistance in marine-derived lineages in freshwater systems. Third, inducible fibrosis was strongly associated with lake characteristics, indicating that ecological context shapes immune strategy. These trait–environment relationships suggest an adaptive basis for divergence in fibrosis among nearby populations. Overall, constitutive immune variation represents an underappreciated axis of population differentiation, whereas inducible variation is more tightly linked to environmental conditions, illustrating how host genetics and ecology jointly structure immune traits in the wild.

## Materials and methods:

### Field survey of fibrosis and infection

To determine the severity and frequency of the fibrosis phenotype and tapeworm infection prevalence in wild stickleback populations, we sampled lake stickleback populations from three geographic regions on Vancouver Island, British Columbia, Canada; 1) the northern region near Port McNeill, 2) the mid-eastern region near Campbell River, and 3) the west coast region near Bamfield, from lakes listed in Table S1. From May 26 - July 3, 2023 we sampled stickleback from 46 lakes on Vancouver Island using a combination of seining and unbaited minnow traps. For stickleback from each lake, we quantified the average fibrosis severity using 10–20 (see Table S1 for exact sample sizes) individuals euthanized and dissected immediately after collection. Fibrosis was scored on a 4-point scale, from 0 (no fibrosis), 1 (some fibrosis, organs do not move freely), 2 (fibrosis, organs adhere together), 3 (organs adhered together and to the peritoneal wall), and 4 (severe fibrosis, difficult to open peritoneal cavity) (Hund et al., 2022). Then, we calculated tapeworm infection prevalence using up to 150 additional stickleback collected from each lake that were stored in ethanol. The sample sizes for fresh dissections and ethanol dissections are included in Table S1. The collections were approved by the Ministry of Forest, Lands, and Natural Resources Operations and Rural Development (Collections permit no. NA23–787881), Fisheries and Oceans Canada (License no. 139753), and the Huu-ay-aht First Nations (Heritage investigation permit no. 2023–013). The sampling sites are located within the traditional territories of the Kwakwaka’wakw and Nuu-chah-nulth First Nations. All collections and experimental activities were approved by University of Connecticut IACUC protocol A21–025.

To obtain measurements of lake characteristics, we used an EXO2 multi-probe sonde (YSI Incorporated, Ohio, USA) to measure temperature-depth profiles at one-meter increments to approximately one meter above maximum lake depth. The sonde could measure to a maximum depth of 20 m, and profiles were taken at or near the deepest point of each lake when possible. We also collected multiple water quality metrics including pH, dissolved oxygen (mg L-1; also calculated as % saturation), and chlorophyll *a* (a proxy for phytoplankton biomass). Measurements for phytoplankton provide insight into qualitative differences among lakes and are units of relative fluorescent units (RFUs). Surface temperature was quantified from measurements at the lake surface (less than 5 meters deep), whereas other sonde variables were summarized as averages across all sampled depths.

We obtained lake bathymetric data using iMapBC, an interactive mapping platform maintained by the Government of British Columbia. To extract bathymetry data for each of the focal lakes, we applied specific map layers within the platform, including Bathymetric – 7.5M (under “Base Maps”), and Lake Bathymetric Maps and Digital Bathymetric Maps (under “Fish, Wildlife, and Plant Species”). These layers provided standardized measurements for each sampled lake, including perimeter (m), maximum depth (m), mean depth (m), surface area (ha), and elevation (m).

### Common-garden experiments

In order to determine which factors contribute to variation in wild stickleback fibrosis, we raised stickleback from multiple populations in the lab to minimize environmental variation and eliminate prior exposure to tapeworms. We then tested their fibrosis responses to several immune challenges, including exposure to live tapeworms, injections of tapeworm proteins from different geographic origins, alum injections, and saline injections.

#### Common-garden: animal breeding and rearing

To identify stickleback populations which exhibit variable infection prevalence and fibrosis, we plotted fibrosis against infection prevalence (Figure 2C) and selected roughly equal numbers of lakes with high fibrosis and low infection, high fibrosis and high infection, low fibrosis and low infection, and low fibrosis and high infections. This resulted in a subset of 26 of the 46 lake populations sampled in 2023 that were subsequently revisited in June 2024. Using unbaited minnow traps and seine netting, we collected gravid females and mature males to obtain gametes for in vitro fertilization to produce 5 –10 full-sib families per population collected under the Ministry of Forest, Lands, and Natural Resources Operations and Rural Development Collections permit no. NA24–89569, Fisheries and Oceans Canada License no. 139753, and the Huu-ay-aht First Nations Heritage investigation permit no. 2024–042. Gametes for crosses were obtained following the Stickleback IVF Breeding protocol (Spence-Jones, 2024). The fertilized eggs were transported to the Bamfield Marine Sciences Center (BMSC), to be reared in aquaria (BMSC AUP #RS-22–09).

Once stickleback larvae had consumed their yolk sac, fry were fed live *Artemia* nauplii. When juveniles reached >1.5 cm in length, they were transferred from 3 L Z-hab tanks to 38 L aquaria supplied with sponge filters and artificial plants for enrichment. Fish were fed twice daily with live *Artemia*, and once they reached ~2 cm in length their diet was supplemented with a ground mixed invertebrate and commercial feed following Stickleback Feeding Protocols (Valentine, 2023), along with suspended decapsulated *Artemia* eggs.

We housed the stickleback in an aquarium system with automated flow-through, supplied with fresh water from the Bamfield Water System and dechlorinated using a Waterite^™^ Excelflow 2472 system (hereafter referred to as “system water”). The light regime was maintained at 16:8 h (light:dark). Because we could not actively control water temperature, it varied seasonally from 15.0 °C to 7.5 °C. Initially, we kept families in the same tank but as the fish grew in size, we occasionally split families among tanks to prevent overcrowding. Fish were maintained under these conditions throughout the experiment.

Once the lab-raised common garden fish were grown, each family was split between treatments to evaluate their fibrosis response, some individuals being exposed to live tapeworms, others injected with antigens to test responses to different immune stimulants, and others were kept as controls.

#### Common garden A: live-tapeworm exposure

To test for population-level differences in infection-induced fibrosis, constitutive fibrosis, and infection rates in lab-raised stickleback, we exposed fish from each population to live tapeworms and quantified fibrosis in exposed fish and unexposed controls. To test for effects of parasite genotype, we used two tapeworm types: Echo Lake tapeworms and Echo Lake × Skogseidvatnet Lake F2 hybrid tapeworms. Tapeworm eggs were generated via a lab-based crossing method described in Weber (2017). After collection, eggs were stored in water at 4°C for at least 4 months. To induce hatching, we distributed eggs across 24-well plates with ~1mL of water in each well, incubated plates in dark incubators at 18°C for 1 week, and then exposed plates to 18:6 light:dark cycles at room temperature. Once hatching began (generally within 1–2 weeks after light exposure), we isolated batches of the cyclopoid copepod *Acanthocycops robustus* (~50 individuals per batch) in ceramic bowls and fasted them for 24 hours. *S. solidus* exposures were performed by pipetting coracidia into the copepod bowls. Although we did not precisely count the number of coracidia, we aimed for >2 tapeworms per copepod (i.e., >100 coracidia per bowl). After 1 week we sampled ~5 exposed copepods per bowl, anesthetized them in carbonated water, and scanned individuals under a dissection microscope to confirm infection presence. The remainder of the experiment was carried out using copepod cups that were at least 2-weeks post exposure and contained at least one confirmed infection.

We exposed lab-raised stickleback (~6 months old) to one of three treatments: unexposed controls, native Echo Lake tapeworms (Vancouver Island), or F2 hybrid tapeworms from Echo × Skogseidvatnet Lake (sample sizes in Table S1). Each fish received four copepods drawn from the exposure cups (individual copepods were not screened for infection prior to feeding), and we recorded the number of copepods consumed by each fish; most fish consumed 3–4 of 4 copepods, with no differences among treatments. We maintained the fish for 80 days after exposure before euthanizing them. At that time, we measured each fish’s mass and length (from the caudal peduncle excluding the tail fin to the tip of the lip), scored fibrosis using the 0–4 scale described above, and for exposed fish, recorded the number of encapsulated and free-living tapeworms.

#### Common garden B - Injections

Next, we tested for population-level differences in fibrosis response to an immune stimulant, fibrosis response to tapeworm protein, and fibrosis response to different tapeworm genotypes. Fish from each population were injected with one of four inoculants: 1) endotoxin-free phosphate-buffered saline (PBS) as an injection control; 2) 1% alum solution (10μL of 2% Alumax Phosphate OZ Biosciences and 10μL of endotoxin-free PBS); 3) 0.5 mg/mL tapeworm protein extract. Within each population, half of the individuals given tapeworm protein were exposed to local tapeworms from the same geographic region as their native lake (e.g., northeast, mid-east, or southwest Vancouver Island); the other half received foreign tapeworm protein from a different region of Vancouver Island. Sample sizes are included in Table S1.

To obtain tapeworms, we sampled fish from three geographical regions: Anutz (anu) from the Port McNeill area, Boot (boo) from the Campbell River area, and Frederick (fre) from the Bamfield area. We euthanized fish in MS-222 and froze them at −20°C until dissections. We extracted tapeworms from the body cavity and homogenized them in 0.9x endotoxin-free PBS using a bead beater and 3.2 mm chrome steel beads, then centrifuged them at 4000 rpm for 20 minutes at 4°C to create tapeworm protein. We measured protein concentration using a RED 660 kit (G Biosciences) and Perkin Elmer Victor^™^ X Multilabel Plate Reader. The protein extract was then diluted to 0.5 mg/mL with 0.9x endotoxin-free PBS. We stored the protein extracts at −80°C for later use. Local and foreign tapeworm protein solutions for injection were prepared by mixing equal quantities of protein extracted from each of 4–5 individual tapeworms from the same lake. Syringes were prepared in a sterilized laminar flow hood no more than one day in advance of injection and refrigerated at 4°C before use.

Prior to injection, we lightly anesthetized fish in 50 mg/L neutral-buffered MS-222. We clipped pelvic or dorsal spines to identify treatments and the experimental period of the injections did not overlap with the experimental exposures, so markings were shared among experiments but not used at the same time. We then injected 20μL of one of the four test inoculants into the left side of the peritoneal cavity following the procedure in (Hund et al., 2022). The needle was inserted at a shallow angle to avoid injury to organs and successful injection was confirmed by observing the distension of the peritoneal cavity. For the duration of the procedure (<1 minute), the fish were placed on a damp sponge with the gills covered in a wet paper towel to minimize stress. After the procedure, we immediately transferred fish to an aerated recovery tank for at least 10 minutes. No fish mortality was observed as a result of experimental injections. Fish assigned to the alum treatment and scheduled for dissection 2 days post-injection were separated into 38 L tanks, while all other treated fish (scheduled for dissection at day 7) were returned to their original tanks. Since families receive every treatment and families are housed together, fish of the same family receiving different treatments are housed together.

Fish were euthanized at either 2 or 7-days post-injection depending on treatment. All fish receiving PBS and tapeworm protein injections were dissected on day 7, while half the fish receiving alum were scored on day 2 and the other half were scored on day 7. During dissections, we measured mass and length, and fibrosis on the 0–4 scale.

#### Quantification and statistical analysis

##### Do infection rates and fibrosis vary among stickleback populations in nature?

We calculated population level prevalences of *S. solidus* by calculating the proportion of individuals who were infected with a detectable tapeworm, for each lake. To confirm that infection prevalence differs among wild-caught samples from the 46 lakes sampled in 2023, we used a binomial generalized linear mixed model (GLMM) in which *S. solidus* infection presence/absence depends on a random effect of the lake. This analysis used a total of 3,321 stickleback sampled from the wild (46 populations with an average of 72 fish per lake).

Fibrosis was scored as a ranked (ordinal) variable ranging from 0 (no fibrosis) to 4 (most severe fibrosis). As a heuristic approach to quantifying population level variation, we calculated the average ordinal score for fish from each population, with standard error confidence intervals. We used a correlation test to evaluate whether mean fibrosis (at the lake level) covaried with infection prevalence, using lakes as the level of replication.

The distribution of residual variation in fibrosis scores violates assumptions of most frequentist linear regression methods. Therefore, to draw statistical inferences about among-population differences in fibrosis we resorted to an Ordinal Logistic Regression method involving a Bayesian hierarchical linear model framework, as described in McElreath (2020). This approach models each increase in fibrosis score above the preceding fibrosis value, as a set of logistic curves, and estimates the log-odds-ratio (lor) that these increase rates differ among populations, or as a function of some specified treatment. For this analysis, fibrosis scores were shifted into ranks 1–5 to fit software requirements. The Bayesian Ordinal Logistic Regression (BayesOLR) involved modeling an individual’s fibrosis score as an ordered logit function with two sets of parameters: a vector of baseline probabilities of the ranks (‘cutpoints’,) and an log-odds-ratio (lor, represented with the symbol ψ) score representing the magnitude of upward transitions through the ranks (e.g., an effect size). That is:

$${\text{Fibrosis}}_{i}\sim \text{dordlogit}(\psi ,\text{cutpoints})$$

The effect size ψ is then modeled as a linear function of independent variables whose effects we wish to test. For example, to test for among-lake variation in fibrosis for wild-caught fish, we test a model in which:

$$\psi =\beta +{\text{lake}}_{i}$$

Where β represents the same-wide fibrosis log-odds-ratio (lor), and ${\text{lake}}_{i}$ is a deviation above or below this mean for any given lake i. The overall mean fibrosis is modeled with a normal prior probability distribution with mean = 0 and standard deviation 2 (e.g., β~norm(μ=0,σ=2)). The distribution of ${\text{lake}}_{i}$ effects is modeled as a normal distribution with a mean of zero and a standard deviation that is an estimated parameter: ${\text{lake}}_{i}\sim \text{norm}\left(\mu =0,\sigma =\sigma_{\text{pop}}\right)$. The prior probability distribution for $\sigma_{\text{pop}}$ is an exponential distribution (with 0 as a lower bound) with mean of 1 ${(\sigma }_{\text{pop}}\sim \text{exp}(1))$. If there is compelling evidence for among-population variation in fibrosis, then the standard deviation $\sigma_{\text{pop}}$ should have a large estimated value, a 95% credibility interval well above zero, and the posterior probability distribution should be shifted towards positive values with low probabilities for $\sigma_{\text{pop}}$ values near zero. Note that because $\sigma_{\text{pop}}$ is bounded to be zero or greater, estimates of $\sigma_{\text{pop}}$ will always be positive, so the key question is whether the magnitude of $\sigma_{\text{pop}}$ is large and its posterior probability distribution gives little support to values near zero. We analyzed this model using the *rethinking* package (McElreath, 2024) in R Studio version 2024.12.1+563 (R Core Team, 2024), estimating parameters with a Monte Carlo Markov Chain algorithm implemented in the *ulam* function with 4 chains replicated on 4 cores for a million samples each. The posterior probability distributions of key parameters were obtained using the *precis()* and *extract.samples()* functions in the *rethinking* package. The cutpoints are modeled with a prior probability distribution that is a normal with mean zero and standard deviation of 1.5. For comparison with this BayesOLR approach, we also used a mixed effects binomial GLMM to test for significant among-population variation in fibrosis, using a frequentist approach. To apply a GLMM we converted the more detailed ordinal scores into a simple presence/absence based on a threshold value of fibrosis, absent if a score of zero, present if a score is 1 or higher. This binomial GLM approach loses resolution because of the thresholding. So, although it yields equivalent results to the BayesOLR, we focus on reporting the Bayesian results.

##### Is there among-population variation in baseline fibrosis in lab-raised stickleback?

We used a BayesOLR to estimate the magnitude of among-population variation in fibrosis in fish that were not subjected to any immune challenge, not injected with PBS, nor exposed to any tapeworm. This model is the same as the one described above for wild caught fish, with the same prior probability distributions. The specific model used is:

$${\text{Fibrosis}}_{i}\sim \text{dordlogit}\left(\psi ,\text{cutpoints}\right)$$

$$\psi =\beta +{\text{lake}}_{i}$$

We used a Pearson correlation to test whether baseline fibrosis population means were correlated with population mean fibrosis in nature. For this analysis, lake represents the level of statistical replication and the data are population estimates no longer restricted to discrete ordinal values. These estimates should have a roughly normal distribution because of the Central Limit Theorem and so are analyzed with frequentist correlation tests. Likewise, we used a Pearson correlation to confirm that population mean fibrosis in uninjected controls was correlated with population mean fibrosis in saline injected fish (e.g., is population baseline fibrosis similar across two different types of controls).

##### Is there among-population variation in fibrosis response to alum in lab-raised stickleback?

We used a BayesOLR to evaluate a statistical model where fibrosis depended on population (a random effect), a general response to alum injection (fixed effect), and a population by injection interaction (a random effect). Specifically, we used MCMC (as described above) to search through parameter space to estimate terms in the following model:

$${\text{Fibrosis}}_{i}\sim \text{dordlogit}(\psi ,\text{cutpoints})$$

$$\psi =\beta +{\text{lake}}_{i}+\beta_{\text{Inject}}*\text{Injection}+{\text{R}.\text{lake}}_{i}*\text{Injection}$$

Where Injection is a binary indicator (0 for PBS injected control fish, 1 for alum-injected fish), and ${R.\text{lake}}_{i}$ estimates the random effect of among-population variation in response to injection (e.g., the population * injection interaction term). For this model we specified the following distributions:

$${\text{lake}}_{i}\sim \text{norm}(\mu =0,\sigma =\sigma_{\text{pop}})$$

$${\text{R}.\text{lake}}_{i}\sim \text{norm}(\mu =0,\sigma =\sigma_{{\text{Inject}}^{*}\text{pop}})$$

and the following priors:

β~norm(μ=0,σ=2)

$$\beta_{\text{Inject}}\sim \text{norm}(\mu =0,\sigma =2)$$

$$\sigma_{\text{pop}}\sim \text{exp}(1)$$

$$\sigma_{\text{Inject}*\text{pop}}\sim \text{exp}(1)$$

The effect size estimate $\beta_{\text{Inject}}$ provides a measure of the magnitude and direction of the stickleback response to alum injection (relative to PBS injected controls), regardless of source population. The random effect estimate $\sigma_{\text{Inject*pop}}$ provides a measure of the magnitude of among-population variation in the response to alum injection. Here we are interested in estimating both the overall response effect size and direction $\beta_{\text{Inject}}$, and whether $\sigma_{\text{Inject*pop}}$ clearly exceeds zero. We ran this analysis three times with different subsets of the data. First, using stickleback that were dissected at day 2 post injection. Then, we reran the analysis using stickleback that were dissected to measure fibrosis at day 7 post injection. Finally, we ran the analysis replacing the binary *Injection* term with a quantitative variable representing the days post-injection to measure the daily rate of response (using PBS as day 0 baseline, and day 2 and day 7 in a single analysis). We used correlation tests to evaluate whether estimated population-specific responses to Alum at day 2, were correlated with estimated population responses to Alum at day 7.

##### Do lab-raised stickleback populations exhibit different fibrosis responses to tapeworm protein?

We next considered a subset of the data including lab-raised fish injected with PBS (a control), or injected with local tapeworm protein, or foreign tapeworm protein. Our first questions were whether fibrosis increases in response to tapeworm proteins (regardless of source) compared to PBS controls, and whether this response varies among stickleback populations. To answer this question we used a BayesOLR with the same model structure as described above for Alum (e.g., $\psi =\beta +{\text{lake}}_{i}+\beta_{\text{Inject}}*\text{Injection}+{R.\text{lake}}_{i}*\text{Injection}$), but now using *Injection* as an indicator variable contrasting control fish (Injection=0) versus fish injected with either tapeworm protein (Injection=1). We sought to estimate the overall response effect size and direction $\beta_{\text{Inject}}$. If the posterior probability distribution for $\sigma_{\text{Inject*pop}}$ clearly exceeds zero, we would infer that lab-raised populations differ from each other in their response to tapeworm antigens.

To determine if stickleback respond more strongly to native than to foreign tapeworm antigens, we used a BayesOLR similar to the one described above. But now we set the *Injection* indicator variable to contrast fish receiving foreign tapeworm protein (Injection=0) versus fish injected with local tapeworm protein (Injection=1). A positive $\beta_{\text{Inject}}$ would indicate a stronger response to local than foreign tapeworms (or vice versa for negative estimates). If $\sigma_{\text{Inject}*\text{pop}}$ clearly exceeds zero we infer there are population differences in response to local versus foreign parasites (e.g., a genotype by genotype interaction effect).

##### Do lab stickleback populations exhibit different fibrosis responses to tapeworm exposure?

When analyzing the outcome of controlled exposures to live tapeworms in laboratory raised fish, we used three analyses asking inter-related questions. First, we separately analyzed the fibrosis response to exposure to Echo Lake tapeworms, and explore to F2 hybrid tapeworms. Using the same priors and distribution assumptions as described for injection experiments above, we fit a BayesOLR model:

$$\psi =\beta +{\text{lake}}_{i}+\beta_{\text{Infect}}*\text{Infection}+{\text{R}.\text{lake}}_{i}*\text{Infection}$$

here *Infection* is a binary indicator (0 for unexposed control fish, 1 for fish that were fed a controlled dose of tapeworm infected copepods). As before we wish to estimate the magnitude and direction of the effect of exposure $\left(\beta_{\text{Infect}}\right)$, and whether this effect varies among stickleback populations $\left(\sigma_{\text{Infect}*\text{pop}}\gg 0\right)$. We did this analysis for Echo Lake tapeworm exposures, then repeated it for hybrid tapeworm exposures. We did not combine these into one analysis because the two tapeworm categories involved different numbers of copepods per individual (e.g., different force-of-infection). However, we did test whether population specific responses to tapeworm exposure were correlated between the two datasets. We also ran a third BayesOLR testing whether fibrosis response differed between fish exposed to Echo Lake versus hybrid tapeworms, and whether this difference varied among stickleback populations. For this third analysis the *Infection* term is a binary indicator (0 for Echo Lake exposures, 1 for hybrid exposures).

##### Do laboratory measures of fibrosis response covary with lake ecological variables?

To determine if constitutive or inducible fibrosis is correlated with environmental covariates, we first conducted a principal component analysis using average lake temperature less than 5 meters deep, average lake pH, average lake chlorophyll *a*, log10 max lake depth, log10 lake area. We then used a Pearson correlation to determine if environmental PC1 was correlated with either constitutive or inducible fibrosis. In order to further test which elements of fibrosis (constitutive, inducible, wild, etc) and tapeworm exposure were associated with environmental covariates, we conducted a canonical correlation analysis between tapeworm/fibrosis associated variables and environmental variables. The tapeworm and fibrosis associated variables included mean fibrosis in wild fish, infection prevalence in wild fish, baseline fibrosis, fibrosis response to PBS, fibrosis response to live tapeworms, fibrosis response to local tapeworms, fibrosis response to foreign tapeworms, fibrosis response to alum on day 2 and day 7, and the average number of tapeworms encapsulated in granulomas. While the environmental variables included were the same that went into PC1. We used a permutation test to determine which of the 6 pairs of canonical variables generated were significant.

Sample sizes for all statistical tests are provided in Table S1 and other statistical parameters are available in the results section. All statistical analyses were performed in the R statistical environment (R Core Team, 2024), using the *ggplot2* (Wickham, 2009) package for plotting. All data and R scripts are available on a data repository (10.5281/zenodo.19824354).

## Resource Availability:

### Lead contact

Further information and requests for resources and reagents should be directed to and will be fulfilled by the lead contact Emma Choi, emma.choi@uconn.edu.

### Materials availability

This study did not generate any unique reagents.

### Data and code availability

- Dissection data from the field survey and experiment have been deposited at Zenodo as 10.5281/zenodo.19824354 and are publicly available as of the date of publication.
- All original code has been deposited at 10.5281/zenodo.19824354 as of the date of publication.
- Any additional information required to reanalyze the data reported in this paper is available from the lead contact upon request.
